# The Relationship between Resistant Hypertension and Advanced Glycation End-Product Levels Measured Using the Skin Autofluorescence Method: A Case–Control Study

**DOI:** 10.3390/jcm12206606

**Published:** 2023-10-18

**Authors:** Tezcan Peker, Bedrettin Boyraz

**Affiliations:** Cardiology Department, Medicalpark Hospital, Mudanya University, Bursa 16200, Turkey

**Keywords:** resistant hypertension, advanced glycation end products, skin autofluorescence

## Abstract

Resistant hypertension is hypertension that cannot be controlled despite the use of three antihypertensive drugs, one of which is a diuretic. Resistant hypertension often coexists with advanced age, obesity, smoking, and diabetes. Advanced glycation end products (AGEs) are substances that are generated as a result of the glycation of proteins, lipids, and nucleic acids due to conditions such as hyperlipidemia, oxidative stress, and hyperglycemia. There are studies showing the relationships between AGE levels and aortic stiffness, hypertension, and microvascular and macrovascular complications in diabetes. In our study, we examined the relationship between resistant hypertension and AGE levels. Our study was planned as a case–control study, and 88 patients with resistant hypertension were included in the focus group, while 88 patients with controlled hypertension were included in the control group. The AGE levels of the patients were measured using the skin autofluorescence method. AGE levels were found to be significantly higher in patients with resistant hypertension than those recorded in the control group. A significant increase in AGE levels was also observed in patients with resistant hypertension and without diabetes compared with the control group. The levels of AGEs, which can be measured cheaply, noninvasively, and quickly with the skin autofluorescence method, may provide benefits in identifying these patients with resistant hypertension.

## 1. Introduction

Critical elements that affect blood pressure (BP) include the amount of blood in the vessels, cardiac output, and vascular resistance. For the control of BP, it is important that the cardiovascular system, kidneys, endocrine system, and nervous system work together in its etiology. While the kidneys are intensely active in impacting vascular resistance and the amount of intravascular fluid, the cardiovascular system and nervous system play important roles in both vascular resistance and cardiac output. Increases in the amount of intravascular fluid and vascular resistance can occur with hypertension (HT) [1,2,3,4,5]. Untreated HT is the leading cause of cardiovascular, renovascular, and neurovascular diseases, as well as mortality, representing the most common cause of preventable cardiovascular deaths and all-cause mortality. In addition, left ventricular hypertrophy, deterioration in the diastolic function of the heart, arrhythmias, and heart failure may develop due to chronic high BP [6,7]. Since our whole body is connected through a vascular network, both the emergence and side effects of HT closely affect the whole body. The risk factors for HT include male gender, advanced age, smoking, diabetes (DM), obesity, family history, early menopause, sedentary lifestyle, and poor psychosocial and socioeconomic factors [8,9]. HT can be successfully controlled with certain clinically proven drugs and lifestyle changes. Regarding lifestyle changes, patients are advised to participate in sports or other physical activity regularly, reduce sodium in their diet, adopt a Mediterranean diet, reduce alcohol intake, reduce weight, and quit smoking. The basis of drug therapies include angiotensin-converting enzyme inhibitors (ACEIs) and/or angiotensin receptor blockers (ARBs), diuretics, calcium channel blockers, and alpha- or beta-blockers; although these drugs can be used alone, combination therapy is often needed. Often, the first drug prescribed is an ACEI or ARB, followed by the addition of a diuretic, and finally, a calcium channel blocker is added [10,11,12]. A high BP that does not respond to treatment despite the combination of these three drugs is called resistant HT. Resistant HT is defined as high BP that cannot be controlled with the maximum dosages of three drugs, one of which is a diuretic. Its frequency is observed in <10% of HT patients. The risk factors for resistant HT include advanced age, obesity, and excessive dietary sodium intake. Resistant HT is often accompanied by left ventricular hypertrophy, DM, atherosclerotic vascular disease, and aortic stiffness. The first steps in treatment include applying stricter lifestyle changes and increasing medication compliance. The recommended treatment for resistant HT is the addition of low-dose spironolactone to existing treatments, or the addition of further diuretic therapy, if the patient is intolerant to spironolactone, with either eplerenone, amiloride, higher-dose thiazide/thiazide-like diuretics, or a loop diuretic, or the addition of bisoprolol or doxazosin [13,14,15]. One reason for this is that renal tubular sodium absorption plays a key role in BP regulation. Increased sodium absorption causes increased water retention, resulting in increases in the amount of intravascular fluid and BP. Increased sodium reabsorption from the proximal tubules is increased in patients with obesity, metabolic syndrome, and DM [2,16]. The compliance of the vessel has an effect on BP; vascular tissue, when a vessel is examined histologically, contains an innermost endothelial layer. The first task of the endothelial layer is to ensure the flow of blood over the smooth surface. Its second task is to regulate both tissue nutrition and vital functions, such as coagulation, by secreting certain substances. The most important of these is endothelial nitric oxide (NO). There is a connective tissue layer just below the endothelial tissue; the task of this connective tissue layer is basically to support the endothelial tissue. Defects in the collagen structure of the connective tissue can cause a decrease in vascular compliance. Outside this layer, there are a muscular and an adventitia layer; the muscular layer is thicker in arteries and adjusts the diameter of the vessel. Both the connective tissue layer and the muscle layer of the vessel play roles in the endothelium and affect blood pressure. Vascular stiffness occurs due to deterioration in the structure of the connective tissue and the thickening of the muscular layer [6,17,18].

Advanced glycation end products (AGEs) are substances that are formed as a result of the glycation of proteins, lipids, and nucleic acids via the Maillard reaction in DM, hyperlipidemia, and oxidative stress conditions [19,20]. The first step of the Maillard reaction takes place between the carbonyl group of a sugar and the amino-terminal group of a protein, lipid, or nucleic acid, but the carbonyl group does not have to come from carbohydrate metabolism—it can also be released during the degradation of proteins or lipids. In addition, it can enter the body exogenously, especially as a result of the consumption of animal-derived foods in a Western-type diet, heating foods, and smoking. In conditions such as renal dysfunction, AGE accumulation in the body increases due to decreased renal clearance [21,22,23]. AGEs demonstrate their undesirable effects by increasing cardiac stiffness, vascular stiffness, endothelial dysfunction, and sodium reabsorption from the renal proximal tubules. As a result, there are increases in extracellular volume, salt, and water retention, which contribute to HT. AGEs are believed to be responsible for increasing the cross-links between collagen molecules. Due to this increase in cross-linking between collagen molecules, there is a decrease in the elasticity of the collagen, which leads to a decrease in the elasticity of the vessel wall and, consequently, to an increase in vascular stiffness. In addition, there are studies suggesting that there is a decrease in NO secretion, secondary to the accumulation of AGEs in the endothelium and that NO utilization decreases. Depending on the decrease in the release and bioavailability of NO, a decrease in vascular stiffness can be observed. It has been shown that AGE levels increase under conditions such as advanced age and resistant HT accompanied by DM [24,25,26,27]. Considering that the main problems in resistant hypertension are the increase in the amount of extracellular fluid and the increase in vascular stiffness, it would not be unreasonable to hypothesize that the condition may be related to AGE levels. Several studies have shown that ARBs reduce AGE levels, and these results indicate that AGE levels may affect HT. For these reasons, we compared the AGE levels in people with resistant HT with those in HT patients.

## 2. Materials and Methods

**Figure 1:**
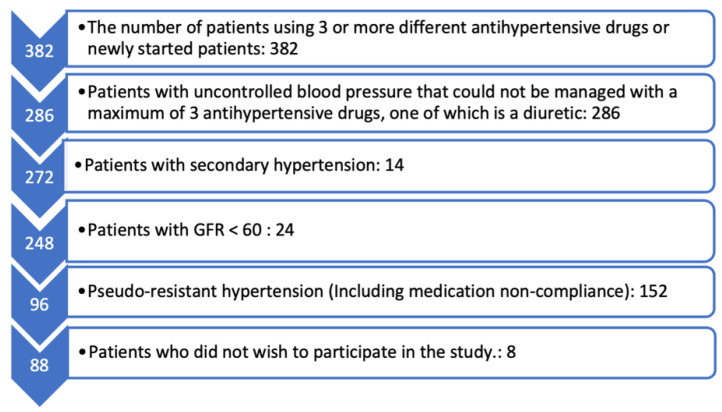
Patient selection.

Our study was designed as a case–control study. Ethical approval was obtained from the ethics committee of Istinye University; the approval number is 23/219. The study was performed in accordance with the Declaration of Helsinki and included 88 patients with resistant HT, with whom we regularly followed up in our hospital, and 88 patients with HT, who comprised the control group. It was assumed that 70 patients per group would be necessary to detect a risk ratio of 2.64 for resistant hypertension, with a power of 80% and p-α of 5%. The patient selection process is illustrated in Figure 1.

Demographic information about the patients, body mass indexes (BMIs), drug treatments used for HT, and antidepressant drug usage were recorded. Patients with a glomerular filtration rate (GFR) < 60 with chronic renal failure, patients with liver failure, and those with end-stage malignancy were not included in the study. Patients with secondary HT and drug incompatibility were also excluded from this study. The exclusion criteria are summarized in Table 1.

The AGE values of the patients were measured by taking the average of three consecutive measurements from the volar side of the right arm with an AGE reader™ (DiagnOptics Technologies B.V., Groningen, The Netherlands) device. Care was taken to measure normal skin sites. Although measurements were performed by the study physician, who was aware of the allocated treatment, the measurement technique does not allow for manipulation of the results. There are numerous studies comparing the skin autofluorescence (SAF) method with invasive measurements. These studies show that the measurements taken using the SAF method are consistent with the data obtained from skin biopsies. Meerwaldt et al. demonstrated a correlation between the AGE values measured through skin autofluorescence and those obtained from skin biopsies in 46 diabetic patients in their studies [28]. Additionally, in their published meta-analysis, Cavero-Redondo et al. argued that the AGE levels measured using the skin autofluorescence method could predict cardiovascular mortality [29]. Furthermore, Meerwaldt et al., in their subsequent study, provided evidence indicating that SAF levels are a strong predictor of cardiovascular mortality in diabetic patients [30]. In another study, Januszewski et al. claimed that SAF levels are correlated with both vascular risk factors and circulating AGE levels [31]. Yamagishi et al. stated in their study that SAF levels could be a marker indicating vascular complications in high-risk patients [32]. All of these studies, in addition to other similar studies, support the view that AGE values obtained from skin biopsies and measurements taken from the skin correlate with the values obtained through the SAF method and that this correlation is also reflected in the clinical condition. For all of these reasons, in this study, we sought to use the AGE values measured noninvasively and easily with the SAF method.

For the statistical analysis, the patients were divided into two groups: those with resistant HT and those without. Comparisons were performed accordingly. Additionally, since DM is a disease known to increase AGE values, patients with DM were excluded from the analysis, and a second additional intergroup comparison was carried out. In the statistical analysis, the comparisons between categorical variables were performed using the chi-square test and the Fischer exact test, and the data were expressed as numbers and percentages. For numerical variables, normality and variance analysis were first performed, for which the Kolmogorov–Smirnov test was used. Afterward, the parameters showing normal distribution were compared with Student’s *t*-test, and the nonparametric variables that did not show normality were compared with the Mann–Whitney U test. Numerical variables are summarized as mean ± standard deviation or median and 25–75% interquartile range (IQR). The results were summarized as *p* < 0.05, with a 95% confidence interval. SPSS 23 (IBM Corp. Released 2015. IBM SPSS Statistics for Windows, Version 23.0. Armonk, NY, USA: IBM Corp.) and STATA.17 (Stata Corp. 2021. Stata Statistical Software: Release 17. Stata Corp LLC.: College Station, TX, USA) programs were used for statistical analysis.

## 3. Results

The numbers of patients in both groups were equal, consisting of 88 patients each. In the statistical analysis of the patients, the patients in the resistant HT group were older on average than those in the control group. While female gender dominance was observed in the group with resistant HT, male gender dominance was observed in the control group. DM and hyperlipidemia were observed significantly more frequently in patients in the resistant HT group, and no significant differences were observed in terms of other disease histories or smoking. The BMI values of the patients in the resistant HT group were found to be higher overall. The frequency of atrial fibrillation (AF) was similar in both groups. Although the ejection fraction (EF) values were found to be similarly median, 60% in both patient groups, they were significantly lower in the group with resistant HT. The AGE values of the patients were found to be significantly higher in the group with resistant HT. These results are summarized in Table 2.

When the drug use of the patients was examined, the most frequently used antihypertensive agents in both groups were ACEIs/ARBs. The most commonly used diuretic agents in the resistant HT group were hydrochlorothiazides. The frequency of antidepressant drug use was significantly higher in the resistant HT group (Table 3).

When the patients with DM were excluded from the analysis, and statistical analysis was performed on the remaining patients, 41 patients in the resistant HT group and 68 patients in the control group, nondiabetic patients in the resistant HT group were found to be significantly older, have higher BMI values, and have a higher rate of AF than the control group. The AGE values of the patients in the group with resistant HT were found to be significantly higher than those of the control group (Table 4).

## 4. Discussion

When our study results were examined, the AGE values in patients with resistant HT were found to be significantly higher than those recorded for patients in the control group. When patients with DM disease were excluded, AGE values were found to be significantly higher in patients with resistant HT [33]. These results suggest that AGEs may be an underlying cause in the pathophysiology of resistant HT, or that risk factors for resistant HT may increase AGE values. When considering the mechanisms that may lead to this condition, the physiology of HT should be considered thoroughly. The first mechanism is renal tubular sodium handling, which plays a critical role in HT. Due to the increase in sodium handling in the renal tubules, the amounts of both water and salt in the body increase, which naturally causes an increase in intravascular volume and, consequently, an increase in BP. The main effect of most antihypertensive agents is based on increasing renal water and salt excretion. This mechanism is particularly important in people with resistant HT because it is one of the most implicated mechanisms in resistant HT. Therefore, the treatment guidelines suggest diuretic therapy as the first treatment in addition to the current treatment in people who develop resistant HT. In their study, Huang et al. suggested that AGEs would increase renal tubular sodium reabsorption in people with hypertension [34]. Yao et al. suggested that proximal renal tubule functions may be impaired as a result of a series of reactions to the increased reception of AGE (RAGE) activity in the hyperglycemic state. They attributed the increase after RAGE activation to high-mobility group box 1 (HMGB1) proteins, members of the nuclear protein family. Cellular necrosis begins secondary to the passive release of HMGB1 from the cell nucleus in the renal tubules. Yao et al. hypothesized that the HMGB1 proteins may cause cellular damage following nuclear factor kB (NF-kB) activation after RAGE interaction, and this effect can be suppressed by sodium-glucose co-transporter 2 (SGLT-2) inhibitors; the results of their study support their hypothesis [35]. When the results of these studies were examined, we found that an underlying mechanism of resistant HT may be increased sodium and salt retention, secondary to decreased sodium excretion from the renal proximal tubules. The role of AGEs in this process may be to damage tubular cells. When the mechanisms of HT are examined, a second cause to consider is the concomitance of vascular stiffness and endothelial dysfunction. Both the flow dynamics of the blood in the blood vessels and the resistance of the vascular structure to this flow play important roles in the emergence of HT. When the vascular structure is examined, there is endothelial tissue in the inner layer, a connective tissue layer under it, muscular tissue, and the adventitia in the outermost layer. Endothelial tissue both creates a smooth flow surface and controls vessel width by secreting certain substances. Perhaps the most important of these substances is NO of endothelial origin. NO causes an increase in vessel width, secondary to a decrease in vascular stiffness and an increase in tissue nutrition. After the accumulation of endothelial AGEs, increased reactive oxygen radicals and decreased NO bioavailability occur [36]. All of these mechanisms, and their relationships with AGEs, support the main aim and hypothesis of our study and the results we subsequently obtained.

The demographic risk factors associated with resistant HT include advanced age (>75 years), obesity, DM, and atherosclerotic vascular disease. In this respect, when our study results were examined, our resistant HT group consisted of older patients than those in the control group, which supports the findings of current studies. In the analysis performed after excluding DM patients, age was found to be significantly higher in patients with resistant HT than in the control group. With increasing age, conditions that can cause increased AGE levels, as well as the presence of increased AGE levels, became more prevalent, which supports the results of our study. When examined in terms of obesity, the mean BMI value of the patients in the resistant HT group was 31.5 kg/m^2^, which is classified as obesity; the mean BMI was 29.4 kg/m^2^ for patients in the control group; thus, BMI was significantly higher in patients with resistant HT than in the control group. Similarly, in the analysis performed after excluding DM patients, BMI values were found to be significantly higher in the resistant HT group. Studies show that an increase in the activity of the AGE–RAGE axis can be both a consequence of obesity and a cause of obesity [37,38]. When assessing the frequency of DM disease, more than half (53.4%) of the patients in the resistant HT group were followed as DM patients, whereas about one out of four patients in the control group were followed as DM patients, representing a significant difference between them. DM increases the risks of resistant HT, obesity, and coronary artery disease, in addition to increasing AGE levels. In cases where blood glucose increases, glycation and, accordingly, AGE levels increase. Studies show that both microvascular and macrovascular complications of DM, as well as diabetic retinopathy, increase in DM patients with high AGE levels [39,40,41]. The incidence of coronary artery disease was similar in both groups. The frequency of AF was found to be significantly higher in the resistant HT group in the analysis that excluded DM patients. Diastolic dysfunction occurs in the heart secondary to AF, which can lead to disruptions in the nutrition of tissues and hemostasis of the body. In their study, Zheng et al. showed that high AGE levels trigger the inducibility of AF in experimental mice by activating the p16/Rb pathway [42]. In their study, Bohm et al. showed that AGE levels measured before pulmonary vein isolation in patients undergoing AF ablation are related to long-term prognosis [43]. This suggests that there may be a relationship between resistant HT and AF.

If there is no additional indication or contraindication in the treatment of HT, an ACEI and/or ARB is often the initial treatment, and a diuretic or calcium channel blocker may be added in combination therapy. In the case of uncontrolled hypertension despite the use of these three agents, the addition of a mineralocorticoid receptor antagonist (MRA) or loop diuretic is recommended, and in patients with continued resistance, adding a beta-blocker or doxazosin is common. However, application in daily practice and the additional comorbidities of patients are not always conducive to effective treatment with these medications. In our resistant HT group, the most frequently used agent was an ACEI or ARB (ACEI: 22.7%, ARB: 73.9%), in approximately 96.6% of patients. The ACEI/ARB drug group also stood out as the most frequently used agent in the control group, with approximately 71.6% of patients using it (ACEI: 31.8%, ARB: 39.8%). When examined in detail, ACEI drug usage rates were similar between the two groups, while ARBs were preferred over ACEIs in the resistant HT group. Several studies show that ARBs reduce AGE levels and oxidative stress loads in patients with HT and DM. One such study is that of Oyo et al., who suggested that the Candesartan molecule reduces AGE levels by reducing oxidative stress in type-2 DM patients with essential HT [44]. In another study, the authors suggested that the Irbesartan molecule showed antioxidative, antiapoptotic, anti-inflammatory, antithrombogenic, and antifibrogenic effects in renal proximal tubule cells by suppressing RAGE mRNA production via the disruption of the AGE–RAGE axis; they claimed that the reason for this is that angiotensin-2 increases RAGE m-RNA production [45]. It is noteworthy that the second most frequently used agents in both groups of our study were beta-blockers. We believe that the use of these drugs, which should be preferred as the fourth or fifth course of treatment, is so high due to comorbidities since more than half of our patients in both groups also had coronary artery disease. One reason why its usage was so high in the resistant HT group is that EF was low in this group, and approximately 68% of patients were also being treated for coronary artery disease. In both groups, these drugs were followed by hydrochlorothiazide diuretics, calcium channel blockers, loop diuretics, doxazosin, and nitrates, in order of common usage. Another remarkable factor in drug use was the use of antidepressant drugs at higher rates in patients in the resistant HT group. This suggests that psychosocial factors are among the causes of resistant hypertension. It is clear that psychosocial factors play a clinical role in some patient groups, so this is considered normal from our point of view.

Patients with resistant hypertension often have more comorbid conditions. In existing comorbidities, such as DM, metabolic syndrome, and renal dysfunction, AGE levels tend to rise. Additionally, prolonged resistant hypertension can lead to impairments in renal function and kidney structure as secondary effects. One of the situations in which AGE levels increase the most is in the case of DM. Another is renal dysfunction, in which elevated levels of AGEs may be observed, but which may either manifest or not yet be reflected clinically for these patients. Of course, the additional comorbid conditions in the studied patients may also contribute to an increase in AGE levels.

## 5. Conclusions

Although the incidence of resistant HT is low, it can be a difficult condition to control and treat. The pathophysiology of why HT, which can be successfully controlled in most people, is resistant to treatment in some people is not very clear. Keeping in mind that patients may have resistant hypertension if their AGE levels are high, the condition can be measured quickly, noninvasively, inexpensively, and without prior training. Our study results support this and show that patients with resistant hypertension have significantly higher AGE levels than patients with controlled hypertension.

## 6. Novelty of the Study

There are studies demonstrating the relationship between AGE levels measured using the skin autofluorescence method and cardiovascular comorbidities in both diabetic and nondiabetic patients, as well as their association with vascular stiffness. Our study is the first to show elevated AGE levels in patients with resistant hypertension. While there may not be sufficient evidence to establish a cause-and-effect relationship, this study can shed light on future, more extensive, investigations that can confirm the results.

## 7. Limitations

The limitation of our study lies in the fact that there are differences in parameters such as age, BMI, DM, HL, and EF between the study groups. An attempt was made to include patients with as many similar characteristics as possible in the study, but it was not fully successful, potentially due to the important role of multi-comorbidity in the pathophysiology of resistant hypertension disease. In patients diagnosed with persistent hypertension, the patients were not followed up with any blood tests, survey methods, or medication counting. Clinical follow-up and the information provided by the patients were taken into account in terms of patient medication compliance, which is a limitation of our study.

## Figures and Tables

**Table 1:** Exclusion criteria.

End-stage renal failure (glomerular filtration rate < 60)
Chronic liver failure
Secondary hypertension
Poor adherence to prescribed medicines
Primary hyperaldosteronism
Atherosclerotic renovascular disease
Untreated sleep apnea
Phaeochromocytoma
Fibromuscular dysplasia
Aortic coarctation
Hyperparathyroidism
Using drugs: oral contraceptives, sympathomimetic agents, cyclosporin erythropoietin, steroids, and cancer therapies
Pseudo-resistant hypertension

**Table 2:** Demographic data.

Variable	Resistant Hypertension	Control Group	*p* Value
Number of patients	88	88	
Age	67.5 (60–75) *	61 (51–68)	<0.001
Gender (male)	35 (39.8%) *	48 (54.5%)	0.07
BMI (kg/m^2^)	31.5 (28.5–36.7)	29.4 (26.3–33.1)	0.001
DM	47 (53.4%)	20 (22.7%)	<0.001
HL	70 (79.5%)	56 (63.6%)	0.02
CAD	63 (71.6%)	54 (61.4%)	0.2
Stroke	2 (2.3%)	1 (1.1%)	0.5
HF treatment	12 (13.6%)	7 (8%)	0.3
Smoking	16 (18.2%)	17 (19.3%)	0.5
Atrial fibrillation	11 (12.5%)	4 (4.5%)	0.1
Ejection fraction	60 (50–60)	60 (55–65)	0.01
AGE level	2.8 (2.5–3.2)	2.1 (1.9–2.2)	<0.001
Creatinine (mg/dL)	0.97 (0.78–1.12)	0.95 (0.87–1.1)	0.8
LDL-C (mg/dL)	111 (77.2–124)	100.5 (78–145.5)	0.4
Triglycerides (mg/dL)	128 (104.7–174.2)	140.5 (110.2–185.2)	0.2
HbA1C (%)	7 (6.7–7.2)	7.2 (6.8–7.8)	0.052

Abbreviations: AGEs—advanced glycation end products; BMI—body mass index; CAD—coronary artery disease; DM—diabetes mellitus; HF—heart failure; HL—hyperlipidemia; LDL-C—low-density lipoprotein cholesterol. *: Categorical variables are summarized as numbers (percentages); numerical variables are summarized as median (25–75% interquartile range).

**Table 3:** Patient medications.

Variable	Resistant Hypertension	Control Group	*p* Value
ACEI + ARB	85 (96.6%)	63 (71.6%)	<0.001
ACEI	20 (22.7%)	28 (31.8%)	0.2
ARB	65 (73.9%)	35 (39.8%)	<0.001
Thiazide diuretic	68 (75.9%)	27 (30.7%)	<0.001
CCB	66 (75%)	29 (33%)	<0.001
MRA	18 (20.5%)	1 (1.1%)	<0.001
Beta-blocker	81 (92%)	60 (68.2%)	<0.001
Loop diuretic	21 (23.9%)	1 (1.1%)	<0.001
Alpha-blocker	13 (14.8%)	2 (2.3%)	0.005
Nitrates	28 (31.8%)	7 (8%)	<0.001
Antidepressant drugs	20 (22.7%)	8 (9.1%)	0.01

Abbreviations: ACEI—angiotensin-converting enzyme inhibitor; ARB—angiotensin receptor blocker; CCB—calcium channel blocker; MRA—mineralocorticoid receptor blocker.

**Table 4:** Demographic data without DM patients.

Variable	Resistant Hypertension	Control Group	*p* Value
Number of patients	41	68	
Age	66 ± 10.8 *	61 ±11.8	0.003
Gender (male)	16 (39%)	37 (54.4%)	0.1
BMI (kg/m^2^)	33 (29–36.9)	29.3 (26–32.7)	0.001
Hyperlipidemia	27 (65.9%)	37 (54.4%)	0.3
CAD	23 (56.1%)	36 (52.9%)	0.8
Stroke	1 (2.4%)	1 (1.5%)	0.6
HF treatment	1 (2.4%)	4 (5.9%)	0.6
Smoking	8 (19.5%)	9 (13.2%)	0.4
Atrial fibrillation	6 (14.6%)	3 (4.4%)	0.07
Ejection fraction	60 (55–60)	60 (60–65)	0.1
AGE level	2.7 (2.4–2.9)	2.1 (1.8–2.2)	<0.001

Abbreviations: AGEs—advanced glycation end products; BMI—body mass index; CAD—coronary artery disease; DM—diabetes mellitus; HF—heart failure. *: Results are summarized as mean ± standard deviation.

## Data Availability

Data are available upon a reasonable request from a data access committee, institutional review board, or the authors themselves.

## Abbreviations

AGEs	Advanced glycation end products
ACEI	Angiotensin-converting enzyme inhibitors
AF	Atrial fibrillation
ARB	Angiotensin receptor blocker
BMI	Body mass index
BP	Blood pressure
CAD	Coronary artery disease
CCB	Calcium channel blocker
DM	Diabetes mellitus
EF	Ejection fraction
e-NO	Endothelial nitric oxide
GFR	Glomerular filtration rate
HF	Heart failure
HL	Hyperlipidemia
HMGB1	High-mobility group box 1
HT	Hypertension
IQR	Interquartile range
MRA	Mineralocorticoid receptor blocker
NF-kB	Nuclear factor-kB
RAGE	Receptor of AGE
SGLT-2	Sodium-glucose co-transporter 2
